# Progress in the Study of Natural Antimicrobial Active Substances in *Pseudomonas aeruginosa*

**DOI:** 10.3390/molecules29184400

**Published:** 2024-09-16

**Authors:** Tianbo Si, Anqi Wang, Haowen Yan, Lingcong Kong, Lili Guan, Chengguang He, Yiyi Ma, Haipeng Zhang, Hongxia Ma

**Affiliations:** 1 College of Life Science, Jilin Agricultural University, Xincheng Street No. 2888, Changchun 130118, China; 20220836@mails.jlau.edu.cn (T.S.); 20230908@mails.jlau.edu.cn (A.W.); yanhaowen@mails.jlau.edu.cn (H.Y.); llguan@jlau.edu.cn (L.G.); he@jlau.edu.cn (C.H.); mayiyi@mails.jlau.edu.cn (Y.M.); 2 The Engineering Research Center of Bioreactor and Drug Development, Ministry of Education, Jilin Agricultural University, Xincheng Street No. 2888, Changchun 130118, China; 3 College of Veterinary Medicine, Jilin Agricultural University, Xincheng Street No. 2888, Changchun 130118, China; congwbs@126.com

**Keywords:** *Pseudomonas aeruginosa*, antimicrobial, secondary metabolites

## Abstract

The prevalence of antimicrobial resistance reduces the effectiveness of antimicrobial drugs in the prevention and treatment of infectious diseases caused by pathogens such as bacteria, fungi, and viruses. Microbial secondary metabolites have been recognized as important sources for new drug discovery and development, yielding a wide range of structurally novel and functionally diverse antimicrobial drugs for the treatment of a variety of diseases that are considered good producers of novel antimicrobial drugs. Bacteria produce a wide variety of antimicrobial compounds, and thus, antibiotics derived from natural products still dominate over purely synthetic antibiotics among the antimicrobial drugs developed and introduced over the last four decades. Among them, *Pseudomonas aeruginosa* secondary metabolites constitute a richly diverse source of antimicrobial substances with good antimicrobial activity. Therefore, they are regarded as an outstanding resource for finding novel bioactive compounds. The exploration of antimicrobial compounds among *Pseudomonas aeruginosa* metabolites plays an important role in drug development and biomedical research. Reports on the secondary metabolites of *Pseudomonas aeruginosa*, many of which are of pharmacological importance, hold great promise for the development of effective antimicrobial drugs against microbial infections by drug-resistant pathogens. In this review, we attempt to summarize published articles from the last twenty-five years (2000–2024) on antimicrobial secondary metabolites from *Pseudomonas aeruginosa*.

## 1. Introduction

Natural products are important sources of new drug precursors, and their diverse chemical structures and wide range of biological activities have always been the focus of attention. The sources of bioactive substances mainly include the metabolites of fungi, bacteria, and substances synthesized by plants. Compounds with different properties are used in different fields, mainly including drug development, with anti-cancer, anti-viral, anti-inflammatory, anti-diabetic, and anti-infection properties for humans [1,2,3]. Not only are they used in clinical applications, secondary metabolites produced by microorganisms play important roles in the agricultural drug, cosmetics, and food industries [4,5]. In 1928, Alexander Fleming discovered penicillin from *Penicillium chrysogenum*, previously known as *Penicillium notatum*, which marked a significant shift from plants to microorganisms as a source of natural products [6]. In addition to penicillin, kanamycin, erythromycin, streptomycin, and other common antibiotics were subsequently developed and successfully used in clinical treatment [7,8,9]. Since that time, antibiotics isolated from bacteria have received increasing attention. It also symbolizes that the secondary metabolites of microorganisms gradually enter application to human life [10].

Microorganisms are capable of producing rich metabolic products, and they have a diversity of different functions [11]. Unlike other materials or chemicals, microbes are alive, and bacteria are particularly valued for their unique ability to evolve in a relatively short period of time. Many bacteria produce so-called secondary metabolites [12]. These biomolecules are not essential for the survival of the organism, but their unique chemical structures and interactions with the environment via various biological activities [13,14,15,16] and with natural compounds, such as omega-3 fatty acids, cyclic peptides, antimicrobial peptides, acetyl derivatives, oligosaccharides, and polysaccharides, can exhibit diverse biological functions, enabling their application not only as antifungals and antibiotics but also as anticancer and anti-inflammatory molecules. At the same time, in the field of drug research and development, continuous breakthroughs in microbial genetic engineering have made the advantages of microbial secondary metabolites gradually manifested [17]. Pathogenic bacteria are among the microorganisms that can cause disease, and include bacteria, fungi, viruses, and parasites [18]. They can grow and multiply in humans, animals, and plants, leading to threats to public health, crop diseases, and animal epidemics, among other things, and causing serious losses in economic development. Over the last few decades, antimicrobial use became widespread, not only in hospitals but also in agricultural environments. For more than six decades, antibiotics have remained unbeaten in their ability to control pathogenic disease-causing agents [19]. However, the overuse of antibiotics in medicine and agriculture has made antibiotic resistance one of the most pressing global health issues [20,21,22]. While some researchers continue to modify existing antibiotics as quickly as new antimicrobials are developed, resistance has increased the demand for new derivatives through the optimization of existing molecular scaffolds [23]. Therefore, the discovery and development of antibiotics with novel structural classes is essential. Although a few bacteria and fungi are human pathogens, most do not interact directly with humans and have been found to inhibit some pathogens [21,24,25]. Bacteria are rich in a variety of secondary metabolites, such as pigments, alkaloids, and antibiotics. Owing to their molecular diversity, these natural compounds have a wide range of therapeutic properties and functions in biology [26]. Thus, current natural medicines focus on fighting diseases caused by pathogenic bacteria that can be countered by bacterial extracts and their secondary metabolites. Secondary metabolites from bacteria are important sources of leads for drug development, and microbial secondary metabolites have contributed the most antibiotics in current clinical use [27].

*Pseudomonas aeruginosa* was first identified in 1882 by French microbiologist Carle Gessard, who isolated it from soldiers’ wound infections. *Pseudomonas aeruginosa* is one of the most common bacteria found in soil, water, air, normal human skin, and the respiratory and intestinal tracts, where the most important condition for this bacterium to live is a humid environment. *Pseudomonas aeruginosa* is a non-fermenting gram-negative bacterium. The bacterium is elongated and of different lengths, sometimes in the form of a bulbous rod, linear, or short chain arrangement. One end of the bacterium has a single flagellum with motility. *Pseudomonas aeruginosa* is a specialised aerobic bacterium with a growth temperature range of 25~42 °C and an optimal growth temperature of 25~30 °C; notably, the bacterium does not grow at 4 °C but can grow at 42 °C, so this feature can be used as an important basis for distinguishing it from other bacteria. At the same time, *Pseudomonas aeruginosa* is a conditional pathogen, which is one of the main pathogens of hospital-acquired infections that infect patients with cystic fibrosis, burn wounds, immunodeficiency, chronic obstructive pulmonary disorder (COPD), cancer, and severe infections requiring ventilation, such as COVID-19 [28]. However, *Pseudomonas aeruginosa* can also produce a variety of active substances that can inhibit a variety of plant pathogenic fungi and bacteria [29,30]. *Pseudomonas aeruginosa* can secrete a variety of antagonistic metabolites against a variety of pathogenic bacteria, which can prevent and control a variety of plant fungal or bacterial diseases; such strains are referred to as *Pseudomonas aeruginosa* preventive strains. *Pseudomonas aeruginosa* produces a wide variety of bacteriostatic secondary metabolites, which include mainly ferrophilins, phenazines, garcinia pyriformis, and extracellular polysaccharides, as well as rhamnolipids, which are not produced by other antibiotic-producing *Pseudomonas* strains, and an increasing number of novel antibiotics are being discovered [31,32]. This approach plays a major role in addressing plant disease control and medical and public health safety.

Iron carriers are bacteriostatic metabolites commonly produced by *Pseudomonas aeruginosa* strains. In low-iron environments, iron carriers can chelate trace amounts of iron in the environment and transfer iron to the body through a specific transport system, which not only enables their own growth needs to be met but also reduces the concentration of iron in the environment and inhibits the growth and reproduction of pathogenic microorganisms, which in turn leads to the control of plant diseases [33]. Another important secondary metabolite produced by *Pseudomonas aeruginosa* is phenoxazines. Phenoxazines are a class of nitrogen-containing heterocyclic molecules. Phenazine antibiotics are secondary metabolites produced by many biocontrol strains of *Pseudomonas aeruginosa* and inhibit the growth of plant pathogenic fungi [34,35,36]. Phenazine antibiotics can act as electron carriers to deliver electrons to target cells after they enter the cell and cause toxic death of target cells by increasing the concentration of intracellular superoxide radicals [37]. To date, the main natural phenazine antibiotics that have been reported are phenazine-1-carboxylic acid and its derivatives phenazine-1-carboxamide (PCN), 1-hydroxyphenylazine (1-OH-PHZ) and pyocyanin (PYO) [38,39]. *Pseudomonas aeruginosa* can produce polyketide antibiotics, among which pyoluteorin (Plt) is a typical aromatic polyketide antibiotic that is chemically known as 2,3-dichloro-5- [2′,6′-dihydroxybenzoyl] pyrrole [40]. Another type of secondary metabolite unique to *Pseudomonas aeruginosa* is rhamnolipids, which are structurally diverse and are formed from one or two β-hydroxy hydrophobic fatty acids linked via β-glycosidic linkages to one or two rhamnose molecules. The rhamnolipids produced by *Pseudomonas aeruginosa* can remediate environmental pollution caused by humans, including oil, metals, or other pollutants in soil, water, coastlines, and seabeds [41,42], and rhamnolipids also have good fungal inhibitory activity. The study of *Pseudomonas aeruginosa* defence strains can not only identify more resources of *Pseudomonas aeruginosa* defence strains but also provide a reference for the study of antimicrobial metabolites produced by *Pseudomonas aeruginosa* [43]. The diversification of secondary metabolites of *Pseudomonas aeruginosa* provides great hope for the development of effective antimicrobial drugs and the prevention and treatment of microbial infections caused by drug-resistant pathogens.

This paper reviews the biological activities and applications of *Pseudomonas aeruginosa* extracts, and their secondary metabolites are reviewed. We cover past and present studies describing the use of these biologics in the treatment of tumors, plant diseases, inflammatory diseases, and infections. In addition, we discuss the ability of some natural products isolated from *Pseudomonas aeruginosa* to exhibit antibacterial, antifungal, and antiviral effects and promote plant growth. Our aim is to emphasize the role of *Pseudomonas aeruginosa* extracts and their identified secondary metabolites in the modern pharmaceutical industry and their importance as potential therapeutic agents.

## 2. Biologically Active Secondary Metabolites Produced by *Pseudomonas aeruginosa*

### 2.1. Phenazines Produced by Pseudomonas aeruginosa

Phenazine compounds are one of the important secondary metabolites of *Pseudomonas aeruginosa* and are now used in plant disease control, medicine, and hygiene. Phenazine 1,6-dicarboxylic acid (PDC) is a phenazine metabolite that can be isolated from *Streptomyces* and *Pseudomonas aeruginosa* [44,45,46]. The production of PDC from *Pseudomonas* sp. has been debated for many years, and recently, PDC production has been shown to be possible only in microorganisms living in anaerobic environments [47]. Debdeep Dasgupta et al. [48] isolated phenazine 1,6-dicarboxylic acid (PDC) from *Pseudomonas aeruginosa* HRW.1-S3. It showed a profound effect on both the planktonic and biofilm modes of growth of DSW.1S4. Phenazine 1,6-dicarboxylic acid (1,6-dicarboxylic acid) possesses antimicrobial activity towards both gram-positive and gram-negative bacteria. Additionally, PDC exhibited cytotoxic activity towards MCF7, HeLa, and HT29 cells of breast, cervical, and prostate cancer origins, respectively, (Table 1).

Shanmugaiah, V et al. [49] isolated a strain of *Pseudomonas aeruginosa*, MML2212, from rice inter-roots and purified and characterized the antimicrobial compound phenazine 1-carboxamide, with the molecular formula C_13_H_9_N_3_O, from this strain. An inhibition assay revealed that PCN significantly inhibited the growth of *Rhizoctonia solani* mycelium at a relatively low concentration (5 μM). In the previous study, PCN inhibited *Rhizoctonia solani* growth and the growth of many other fungal phytopathogens [17]. In addition to antifungal activity, the purified PCN of *Pseudomonas aeruginosa* MML2212 exhibited antibacterial activity against *Xanthomonas oryzae pv. oryzae* that was slightly greater than that of the commercial antibiotic rifamycin. This result lays an important foundation for the control of this strain.

Asian soybean rust (ASR), caused by *Phakopsora pachyrhizi* Syd, is one of the most economically important crop diseases [78]. The outbreak caused huge losses to soybean production [79]. Neve et al. [29] isolated a strain of *Pseudomonas aeruginosa* LV from a citrus canker lesion of *Citrus sinensis* cv. Valencia fruit in Astorga, Brazil. The semi-purified fraction F4A was subsequently extracted from the cell-free supernatant via liquid vacuum chromatography (LVC). The purified F4A fraction mainly contains four important metabolites, including phenazine-1-carboxylic acid (PCA), phenazine-carboxamide (PCN), indole-3-one (IND), and an organocopper antimicrobial compound (OAC). Among them, OAC can reduce the infection of Candidatus Liberibacter asiaticus when applied to citrus plants [21]. The concentrations of each compound in the F4A total volume were 30% for PCA, 25% for PCN, and 25% for OAC. In this study, secondary metabolites of *Pseudomonas aeruginosa* LV were used for spore germination and defense gene expression in infected soybean plants. F4A was found to inhibit spore germination, and the organocopper antimicrobial compound (OAC) in F4A contributed to the overexpression of defense-related genes when sprayed on young soybean shoots. More importantly, F4A was found to reduce ASR severity and lesion frequency when applied to plants. A series of results on spore germination, gene expression, disease severity, and disease control indicate that the secondary metabolites of *Pseudomonas aeruginosa* LV have great potential in controlling this critically important fungal disease.

The rhizosphere soil of some plants is also a good source of antagonistic strains. More than 10 years ago, a team from the School of Life Science and Technology of Shanghai Jiao Tong University isolated and screened a batch of *Pseudomonas* M18, GP72, and SJT25 strains with antibacterial activity from rhizosphere soil samples of rice, melon; and pepper [80,81]. Prior to this study, the most common strain, *Pseudomonas aeruginosa* M18, was known to produce at least two bacteriostatic metabolites, shenazinomycin (PCA) and luteolin [82]. Zhou et al. [60] collected healthy rice rhizosphere soil and isolated and screened *Pseudomonas aeruginosa* PA1201, which can inhibit the growth of rice sheath blight. Strain PA1201 was identified by mass spectrometry to produce two inhibitory metabolites, shenazinomycin and phenazine-1-amide. The yield of detenazinomycin in strain PA1201 was 3–4 times greater than that in *Pseudomonas aeruginosa* M18; the metabolic product phenazine-1-amide (PCN), which has greater antibacterial activity, was produced, and the maximum yield of PCN in soybean powder fermentation medium was 489.5 mg/L. According to recent worldwide reports, the production of shenazinomycin and phenazine-1-amide by *Pseudomonas aeruginosa* PA1201 is also outstanding. This provides a new microbial resource for the development of new agricultural antibiotics. Although strain PA1201 is derived from the rice rhizosphere, it still has certain toxicity to human lung adenocarcinoma cell lines and *Drosophila*, so the development and utilization of strain PA1201 must be carried out with high caution.

Xu et al. [57] collected 60 strains of *Pseudomonas aeruginosa* and screened them for their bacteriostatic effects against *Staphylococcus aureus* (ATCC 25923) via filter paper disc, cross-stripping, and coculture methods. Chloroform isolates were tested for their bacteriostatic effects against gram-positive organisms, namely, methicillin-resistant *Staphylococcus aureus* (MRSA), vancomycin intermediately resistant *Staphylococcus aureus* (VISA), and *Corynebacterium* (CS); gram-negative bacteria, namely, *Acinetobacter baumannii* (AB) and *Moraxella catarrhalis* (MC); and fungi, namely, *Candida albicans* (CA), *Candida tropicalis* (CT), *Candida glabrata* (CG), and *Candida parviflora* (CP), were evaluated for their bactericidal activity. The PA06 and PA46 strains were found to have strong antimicrobial activity. Subsequent detection of the main components of the antibacterial activity via high-performance liquid chromatography (HPLC) and electrospray mass spectrometry (ESM) resulted in the identification of four antibacterial compounds. The main presumption was that these compounds were phenazine analogs because of their functional similarity and relatively high molecular weight. However, it was determined that it was not PCA or pyocyanin. The exact structures of these compounds need further analysis, but a diversity of antimicrobial *Pseudomonas aeruginosa* secondary metabolites have been observed.

The ecological diversity of the marine environment increases the abundance of the *Pseudomonas* genus, and the resources of bioactive metabolites are diverse [83,84,85]. Zhang et al. [58] isolated a marine strain of *Pseudomonas aeruginosa* PA31x from sediments in the Yellow Sea of China and reported that the natural product produced by this strain inhibited the growth of *Vibrio anguillarum* C312, one of the most severe pathogens in mariculture. A combination of high-resolution electrospray ionization mass spectrometry and nuclear magnetic resonance (NMR) spectroscopy subsequently demonstrated that the antimicrobial substance was a phenazine derivative, phenylamine-1-carboxylic acid (PCA). To understand the antimicrobial mechanism of PCA against pathogenic bacteria, morphological changes were observed using scanning electron microscopy (SEM) and transmission electron microscopy (TEM), which revealed that the surface of the cell membranes became roughened, with the appearance of one to a few cystic bumps, distortions, and deeper grooves and ripples. Transmission electron microscopy images indicated that the mechanism of bacterial inactivation was mainly through cytoplasmic breakdown. Additionally, PCA revealed effective disease control in tobacco infected with *Phytophthora nicotianae* JM1 via disruption of the mycelia of *Phytophthora nicotianae* JM1. The ability of PCA to generate reactive oxygen species in cells was subsequently demonstrated by the presence of elevated amounts of intracellular ROS in PCA-treated *Vibrio eelensis* and human carcinoma A549 cells. Experiments on the protection of zebrafish embryos by PCA were carried out with zebrafish as the research subject. The percentage of hatched embryos infected with *Vibrio anguillarum* C312 was the lowest among the groups, at only 3.8%. However, the percentage of hatched zebrafish embryos apparently increased to 42.3% and 84.62%, respectively, when 2 µg/mL and 3 µg/mL PCA were co-incubated with *Vibrio anguillarum* C312. Microscopy revealed that zebrafish embryos treated with DMSO and PCA developed normally at 48 h postfertilization (hpf). However, zebrafish embryos infected with *Vibrio anguillarum* C312 developed abnormally and could not mature. These findings together indicate that PCA could weaken *Vibrio anguillarum* infection in zebrafish embryos in the water environment and could be applied as a protective agent in aquaculture production. Taken together, the results of this study revealed not only the in vitro antibacterial and antifungal activities of PCA against several aquatic and plant pathogens but also its in vivo antibacterial and antifungal activities in host animals or plants. These findings lay the foundation for the study of the effectiveness and practicality of PCA in disease control.

Rawi et al. [59] also screened a strain of *Pseudomonas aeruginosa* Rlimb with good bacteriostatic activity from marine microorganisms. The bioactive compounds were extracted from the ester of the cell-free supernatant of this strain via ethyl acetate extraction. The CFS was verified to inhibit the growth of four strains: *Bacillus cereus*, *Streptococcus uberis*, *Pseudomonas* sp., and *Vibrio parahaemolyticus*, via a bacteriostatic assay. Significant changes in biofilm inhibition were subsequently detected via the crystal violet and the aqueous tank tests. Interestingly, the results of both tests revealed that lower concentrations of bacterial cultures were more effective at inhibiting bacterial biofilm formation than higher concentrations of bacterial cultures were, possibly because a higher concentration of bacterial culture solution can promote bacterial adhesion and increase the number of colonies of biofilm-forming bacteria. However, this does not negate the fact that the ethyl acetate extract of the strain proved to be a good antifouling agent, preventing the adhesion of biofilm-forming bacteria. Although this study did not result in the material identification of the ethyl acetate extract of *Pseudomonas aeruginosa*, it was sufficient to illustrate the diversity of antimicrobial secondary metabolites of *Pseudomonas aeruginosa* Rlimb and the potential for their development.

The *Pseudomonas aeruginosa* strain 2016NX1 [86], which has antibacterial and antifungal activity, was isolated from the root of *Millettia speciosa* by TT Liu [63]. The blue and yellow fractions were obtained by extraction and purification of the secondary metabolites of the strain. The main components were identified by NMR as cereusitin A and 1-hydroxyphenazine, respectively. The yellow component (1-hydroxyphenazine) was subsequently verified for its antibacterial activity, and was found to have antibacterial activity against five types of disease-causing bacteria, including *Salmonella* sp., *Klebsiella oxytoca*, *Shigella castellani*, *Salmonella typhi*, and *Bacillus anthracis*, and it also has antibacterial activity against five fungi, including *Cochliobolus miyabeanus*, *Diaporthe citri*, *Rhizoctonia solani*, *Phytophthora parasitica var. nicotianae*, and *Exserohilum turcicum*. Therefore, the isolated strain 2016NX1 has important application prospects in the prevention and control of plant diseases and the protection against pathogenic bacterial infection.

The *Pseudomonas aeruginosa* SD12 strain was isolated from soil contaminated with leather waste by Dharni et al. [73]. The bacterium showed good antibacterial activity against a variety of plant pathogenic fungi, including *Alternaria alternata*, *Bipolaris australiensis*, *Colletotrichum acutatum*, *Fusarium oxysporum*, *Alternaria solani*, and *Colletotrichum acutatum*, and the main bioactive substance was 1-hydroxyphenazine, as determined by nuclear magnetic resonance spectroscopy. The authors also reported that the strain can produce phosphatase, cellulase, protease, pectinase, and HCN and retained the ability to produce hydroxy-acid-type iron carriers, which enhances the potential use of this strain as an effective bioinoculant for soil fertility, plant protection, and the promotion of plant growth with reduced disease incidence.

The ability of *Pseudomonas aeruginosa* ID 4365 to produce pyoverdin types of siderophores makes it a control strain against pathogens from marine-derived peanut plants. Makarand Ramesh Rane et al. [69] revealed previously undetected metabolites in the culture supernatant of this strain rich in pyoverdin, which also played an antibacterial role. In addition to pyoverdin, the strain produced additional siderophores, viz. pyochelin and salicylic acid. It was confirmed that viz. pyocyanin and phenazine-1-carboxylic acid had antibacterial activity against *Aspergillus niger*, *Colletotrichum falcatum*, *Colletotrichum capsicum*, *Fusarium oxysporum*, and *Sclerotium rolfsii*. Iron- and phosphate-dependent coproduction of siderophores and phenazines was also confirmed. An experiment also revealed that *Pseudomonas aeruginosa* ID 4365 can produce hydrogen cyanide and indol-3-acetic acid and solubilize phosphate. This study revealed that *Pseudomonas aeruginosa* is a good biocontrol strain and demonstrated that there are interactions and synergies between metabolites.

A strain of *Pseudomonas aeruginosa*, GC-B26, with strong antifungal and anti-oomycete activity was isolated from Korean grassland soil by Jung Yeop Lee et al. [75] The antibiotic G26A, which is active against *Phytophthora capsici* Leonian and *Colletotrichum orbiculare* (Berk & Mont) van Arx, was isolated from culture filtrates of *Pseudomonas aeruginosa* strain GC-B26 via various chromatographic procedures. The antibiotic G26A was identified as phenazine-1-carboxylic acid by NMR spectroscopy. *Colletotrichum orbiculare*, *Phytophthora capsica*, and *Pythium ultimum Trow* were the most sensitive to G26A, with MIC values of approximately 5 µg mL^−1^. However, it has no antibacterial activity against yeast or bacteria.

### 2.2. Rhamnolipids Produced by Pseudomonas aeruginosa

Structural homologues of rhamnolipids produced in early *Pseudomonas aeruginosa* have been identified. The types of bacterial strains, carbon sources used, and process strategies for rhamnolipid production were correlated [87,88]. E. Haba et al. isolated *Pseudomonas aeruginosa* 47T2 NCIB 40,044 from an oil-contaminated soil sample and purified the new product RL47T2 from the metabolites of this strain [52]. RL47T2 is a new product formed from 11 rhamnolipid homologues (Rha-Rha-C8-C10; Rha-C10-C8/Rha-C8-C10; Rha-Rha-C8-C12:1; Rha-Rha-C10-C10; Rha-Rha-C10-C12:1; Rha-C10-C10; Rha-Rha-C10-C12/Rha-Rha-C12-C10; Rha-C10-C12:1/Rha-C12:1-C10; Rha-Rha-C12:1C12; Rha-Rha-C10-C14:1; Rha-C10-C12/Rha-C12-C10) produced by *Pseudomonas aeruginosa* 47T2 from waste cooking oils. The physicochemical and biological properties of RL47T2 as a new product were also studied. It was found that the surface tension and the interfacial tension with kerosene were in a downward trend. At the same time, the antibacterial ability of the substance is also very excellent, showing good antibacterial activity against many strains, including *Serratia marcescens*, *Enterobacter aerogenes*, *Klebsiella pneumoniae*, *Staphylococcus aureus*, *Staphylococcus epidermidis*, *Bacillus subtilis*, and phytopathogenic fungal species *Chaetomium globosum*, *Penicillium funiculosum*, *Gliocadium virens*, and *Fusarium solani*. Owing to its physicochemical and antimicrobial properties, RL47T2 may be used in bioremediation in the food and agrochemical industries.

Biosurfactants are unique secondary metabolites that are non-ribosomally synthesized by actively growing and/or resting microbial cells (bacteria, fungi, and yeast) [80,89,90]. Ndlovu et al. [55] isolated *Pseudomonas aeruginosa* ST5 from wastewater, followed by solvent extraction and characterization of the biosurfactant compounds via ultra-performance liquid chromatography (UPLC) coupled with electrospray mass spectrometry (ESI-MS). The solvent extracts obtained from the ST5 strain were confirmed to be a mixture of rhamnolipid congeners of mono-rhamnolipids (Rha–C12–C10/Rha–C10–C12; Rha–C10–C10; Rha–C10–C8/Rha–C10–C8) and di-rhamnolipids (Rha–Rha–C12–C10/Rha-Rha–C10–C12; Rha–Rha–C10–C10; Rha–Rha–C10–C8/Rha–Rha–C10–C8). The bacteriostatic activity of the ST5-produced extracts containing rhamnolipid homologs was then determined via an agar disc diffusion assay against various reference, environmental, and clinical bacterial and fungal strains. The results revealed inhibitory activity against all the gram-negative bacteria tested, including *Escherichia coli* (ATCC 417373), *Klebsiella pneumoniae* (P2), and *Salmonella enterica* (SE19). Rhamnolipid extracts also showed strong bacteriostatic activity against gram-positive bacteria, including *Staphylococcus aureus* (ATCC 25923), *Bacillus cereus* (ATCC 10876), and *Bacillus cereus* (ST18), among others. In addition, the rhamnolipid extract showed a considerable zone of inhibition (22.3 ± 0.9 mm) against *Bacillus cereus* ST18. Additionally, the rhamnolipid extract showed broad-spectrum antimicrobial activity against various fungi, including *Cryptococcus neoformans* CAB1063, *Cryptococcus neoformans* CAB1034, and *Candida albicans* 8911, which all have good inhibitory diameters. They demonstrated that biosurfactant-producing strains isolated from wastewater have potential for large-scale production of various analogous congeners of rhamnolipid biosurfactant compounds for use as antimicrobial agents in the medical and food industries.

Sulfate-reducing bacteria (SRB) are important microorganisms that cause iron corrosion on metal surfaces under anaerobic and aerobic conditions, and their corrosion is related to the formation of biofilms [91,92,93]. To address the challenge of eliminating SRB biofilms, Thammajun et al. [67] identified the ability of *Pseudomonas aeruginosa* PA14 supernatant to disperse SRB biofilms. To determine the biochemical basis of SRB biofilm dispersion, the authors examined a series of *Pseudomonas aeruginosa* mutants and found that the rhamnolipid production-defective mutants rhlA, rh1B, rhlI, and rh1R had significantly reduced the SRB biofilm. The *Pseudomonas aeruginosa* supernatant disperses the SRB biofilm via rhamnose lipids. The authors then performed a complete transcriptional analysis (RNA-seq) to determine the genetic basis of *Pseudomonas aeruginosa* supernatant-dispersed SRB biofilms. On this basis, we also identified four proteins (DVUA0018, DVUA0034, DVUA0066, and DVUA0084) that affect biofilm formation in *Desulfovibrio vulgaris* macroplasmids. The production of DVUA0066 (a possible phospholipase) reduces biofilm formation by 5.6-fold. In addition, the supernatant of *Pseudomonas aeruginosa* dispersed the SRB biofilm more easily than the protease in M9 low-glucose medium and had a greater inhibitory effect on the biofilms of *Escherichia coli* and *Staphylococcus aureus*.

### 2.3. Other Active Substances Produced by Pseudomonas aeruginosa

Antonio Ramkissoon et al. [50] reported the first isolation of three indole alkaloid compounds from a Pseudomonad bacterium, *Pseudomonas aeruginosa* UWI-1. They were identified as tris(1H-indol-3-yl) methylium, bis(indol-3-yl) phenylmethane, and indolo (2, 1b) quinazoline-6, 12 dione. The in vitro bacteriostatic activity of these three compounds was evaluated via the broth microdilution technique. Tris(1H-indol-3-yl) methylium and tris(1H-indol-3-yl) methylium displayed antibacterial activity against only gram-positive pathogens, although tris(1H-indol-3-yl) methylium had a significantly lower minimum inhibitory concentration (MIC) value than did bis(indol-3-yl) phenylmethane. Indolo (2,1b) quinazoline-6,12 dione displayed potent broad-spectrum antimicrobial activity against a range of gram-positive and gram-negative bacteria.

Rina Hidayati Pratiwi et al. isolated and purified antibacterial compounds from the *Pseudomonas aeruginosa* strain UICC B-40 [51]. The antibacterial compound (2E,5E)-phenyltetradeca-2,5-dienoate (molecular weight 300 g/mol), which is composed of a phenolic ester, fatty acid, and long chain of aliphatic group structures, was successfully identified in that study. The antibacterial compound designated (2*E*,5*E*)-phenyltetradeca-2,5-dienoate exhibited antibacterial activity against two gram-positive pathogenic bacteria (*Staphylococcus aureus* ATCC 25923 and *Bacillus cereus* ATCC 10876), with a MIC of 62.5 μg/mL for *Staphylococcus aureus*. Scanning electron microscopy revealed that the mechanism of action of the compound involves the breakdown of the bacterial cell wall through lysis.

Bacterial leaf blight (BLB) and bacterial leaf streak (BLS) are caused by *Xanthomonas oryzae pv. oryzae* (Xoo) and *Xanthomonas oryzae pv*, respectively [94,95,96,97]. Sumera Yasmin et al. [43] screened 512 bacterial isolates from rice inter-roots for determinants of plant growth promotion and revealed that *Pseudomonas aeruginosa* BRp3 was the most effective at solubilizing phosphorus in vitro at 97 µg/mL and producing the phytohormone indoleacetic acid at 30 µg/mL and an iron carrier at 15 mg/L. The results of the screening are presented in the following table. Moreover, the strain showed antagonistic effects against a variety of plant pathogens, such as *Xanthomonas oryzae pv. oryzae* (Xoo) and *Fusarium*, among which the BRp3 strain showed consistent inhibitory effects against different strains of rice bacterial leaf blight (BLB) pathogens. Mass spectrometric analysis revealed the production of siderophores (1-hydroxyphenazine, pyocyanin, and pyochelin), rhamnolipids, and a series of previously characterized 4-hydroxy-2-alkylquinolines (HAQs), as well as novel 2,3,4-trihydroxy-2-alkylquinolines and 1,2,3,4-tetrahydroxy-2-alkylquinolines in the crude extract of BRp3. Interestingly, they also used the strain as an inoculant and reported a significant increase in both grain and straw yields in field experiments. These results provide evidence that novel secondary metabolites produced by BRp3 may contribute to its activity as a biological control agent against Xoo and its potential to promote the growth and yield of Super Basmati rice.

The structurally diverse secondary metabolites of *Pseudomonas aeruginosa* have been identified as having inhibitory potential against aquaculture pathogens, particularly *Vibrio* and *Aeromonas* [98,99,100,101]. Anusree V. Nair et al. [53] isolated a strain of *Pseudomonas aeruginosa* Ps04 and evaluated its antibacterial ability against aquatic pathogens. They found the cell-free supernatant (CFS) of *Pseudomonas aeruginosa* Ps04 exhibited strong antibacterial activity against major aquaculture pathogens belonging to the genera *Vibrio* and *Aeromonas*. The research found that the antibacterial substance had good acid–base, temperature, and enzyme stability. The active substance was purified and analyzed by wave spectroscopy and it was finally determined that the compound 4-hydroxy-11-methylpentacyclo [11.8.0.02,3.011,12.016,17] henicosa-1,3,5,8(9),17-penten-14-one is responsible for its major antibacterial activity. The antimicrobial power and stability demonstrated by this new antibacterial compound can be developed as novel antibacterial drugs and could make a significant contribution to aquaculture applications.

An increasing number of scholars are using microorganisms to transform steroid compounds and study the activity of their products [102,103]. To date, more than 300 sterols have been recorded, making them the best-selling drugs, second only to antibiotics [104]. Xiaohe Li et al. [54] used pregnenolone as the only carbon source for the screening medium and sieved soil samples taken from the Wuhan Institute of Science and Technology to obtain a strain of *Pseudomonas aeruginosa* HBD-12, which was found to have strong inhibitory effects on *Escherichia coli*, *Bacillus thuringiensis*, *Penicillium italicum*, and *Penicillium digitatum*, as determined by an inhibitory activity assay. Column chromatography was subsequently used to isolate the secondary metabolites of the strain to obtain two monomeric compounds, which were identified as 1-hydroxy-9,10-diazaphenanthrene for HBD1 and 3-hydroxy-9,10-dihydrodiazaphenanthrene for HBD2, via spectroscopic analysis. The authors used Aurora-A kinase as a screening model for the first time to determine the antitumor activity of these two compounds and reported that both compounds were able to inhibit the activity of AURKA, and the inhibition rate of 1-hydroxy-9,10-diazaphene against AURKA was as high as 78.39% ± 2.29%. However, owing to the low mass of the compounds, the inhibitory activity of the single compounds was not determined.

The endophytes of plants are also a source of *Pseudomonas aeruginosa*, and their metabolic activity gives them the ability to survive in the complex chemical environment of their host and provides a selective advantage in terms of growth improvement and disease resistance [105,106]. Khan et al. [56] isolated a plant-promoting antifungal endophytic bacterial strain, Ld-08, from lily bulbs and identified it as *Pseudomonas aeruginosa*. The isolated strain Ld-08 exhibited antagonistic effects against several fungal pathogens, such as *Fusarium oxysporum*, *Botrytis cinerea*, *Botryosphaeria dothidea*, and *Fusarium fujikuroi*, and inhibited mycelial growth in a dual culture plate experiment. In the present study, the secondary metabolites in the ethyl acetate fraction of the isolated endophytic strain Ld-08 were assayed. Several bioactive compounds and secondary metabolites with potential antifungal properties were identified. The most important bioactive compounds were quinolones [2-(2-hepten-1-yl)-3-methyl-4-quinolinol,2-heptyl-3-hydroxy-4-quinolone,1-methyl-2-nonylquinolin-4-one]; 3,9-dimethoxypterocarpan; cascaroside B; dehydroabietylamine; epiandrosterone; nocodazole; oxolinic acid; pyochelin; rhodotulic acid; 9,12-octadecadienoic acid; di-peptides; tri-peptides; pinolenic acid methyl ester; and urodiol and venlafaxine. Through quantitative assays, strain Ld-08 was subsequently shown to be able to produce organic acids, ACC deaminase, phospholysis, IAA, and iron carriers. Through inoculation experiments, they also reported that Ld-08 had a significant effect on growth parameters such as plant height, leaf length, bulb weight, and the root length of lilies, making it a promising growth promoter. Therefore, the improvement in growth of the inoculated plants might be attributed to the endophytic colonization of *Pseudomonas aeruginosa* Ld-08 with growth-promoting attributes, including antifungal potential, IAA, siderophore production, nitrogen fixation, and phosphate solubilization.

Chemical control is now lacking due to the adverse effects of chemicals on the environment and soil microbiota, which require alternative inputs that are less dependent on chemicals to achieve sustainable agriculture [107,108,109]. Tomato wilt caused by the fungus *Fusarium oxysporum* Schlecht.f. sp. lycopersici (Sacc.) W.C. Snyder et H.N. Hansen (FOL) is an alarming disease, causing yield losses of up to 25% [110,111]. Therefore, the use of biological control via induced systemic resistance (ISR), which uses rhizosphere plant growth to promote bacterial activity against fungal pathogens, is currently important [112,113]. Sabin Fatima and Tehmina Anjum [61] investigated the potential of *Pseudomonas aeruginosa* PM12 to induce systemic resistance to fusarium wilt in tomato. They isolated and identified the bioactive compounds responsible for ISR from sterile cell supernatants. The compounds were identified by gas chromatography/mass spectrometry. The results revealed that the active components of ISR included 3-hydroxy-5-methoxy benzene methanol (HMB), eugenol, and tyrosine. Subsequent bioassays demonstrated that HMB was a potential determinant of ISR and significantly improved tomato wilt when HMB was applied at a rate of 10 mM via the soil drench method. The metabolic dynamics of tomato plants infected with *Fusarium oxysporum* were involved in the defence mechanism, ensuring the activation of ISR by the bacterial stimulator HMB and resulting in strong reregulation of defense-related pathways. HMB is a potential promoter involved in the dynamic reprogramming of plant pathways, and its function contributes to the defense response.

Cystic fibrosis (CF) is an autosomal recessive disease. The fungal pathogens *Aspergillus fumigatus* were isolated from the sputum of patients with CF. *Aspergillus fumigatus* infection is associated with an increased risk of *Pseudomonas aeruginosa* infection, and co- infection studies have demonstrated that *Pseudomonas aeruginosa* may inhibit the growth of *Aspergillus fumigatus* [114,115,116,117]. *Aspergillus fumigatus* can secrete some secondary metabolites such as gliotoxin, which is more advantageous for its survival [118,119,120,121,122]. This may be the root cause of inhibition by *Pseudomonas aeruginosa*. The way one pathogen affects another’s metabolome can have serious consequences for the host, and competition between microbes often leads to altered secretory characteristics and increased damage to host tissues [123,124]. Anatte Margalit et al. [62] studied the effects of *Pseudomonas aeruginosa* culture media on fungal growth and gliotoxin production. When *Pseudomonas aeruginosa* CUF and *Aspergillus fumigatus* CUF were co-incubated, the production of gliotoxin was low in fungal cultures exposed to *Pseudomonas aeruginosa* CUF. These results suggest that the response of *Aspergillus fumigatus* to *Pseudomonas aeruginosa* may be modulated through direct (via bacterial cells) and indirect (via bacterial secretomes) interactions with the bacteria. The secretomes of *Pseudomonas aeruginosa* were analyzed via LFQ proteomics. Two metabolic enzymes, isocitrate lyase and malate synthetase, were detected in *Pseudomonas aeruginosa* CUF. A range of proteins involved in detoxification, including catalase and thioredoxin, key components of the antioxidant system, were also found in the culture filtrate, indicating that the bacterial cells in this growth medium were subjected to an environmental stress response. Subsequently, quantitative proteomic analysis was used to study the proteomic reaction of *Aspergillus fumigatus* after exposure. Proteomic data analysis in this study identified a decrease in the relative abundance of several ribosomal-associated proteins, which was evident in the groups exposed to *Pseudomonas aeruginosa* CUF. The relative abundance of four proteins associated with gliotoxin biosynthesis gene clusters decreased significantly in *Aspergillus fumigatus* that was simultaneously exposed to *Pseudomonas aeruginosa* CUF. However, a significant increase occurred in the relative abundance of proteins associated with the biosynthesis of other secondary metabolites. Proteomic data also revealed that the relative abundance of several proteins associated with mitochondria was reduced. These changes in the proteome suggest that the bacterial secretome has a profound influence on the fungal proteome. Alterations in the abundance of proteins involved in detoxification and oxidative stress highlight the ability of *Aspergillus fumigatus* to differentially regulate protein synthesis in response to the environmental stress imposed by competitors.

*Macrophomina phaseolina* is a fungus that causes dry root rot along with diseases such as stem and root rot, charcoal rot, and seedling blight [125,126,127]. Devaraj Illakkiam et al. [64] reported that *Pseudomonas aeruginosa* PGPR2 protected mung bean plants from charcoal rot disease caused by *Macrophomina phaseolina*. The ethyl acetate extract of *Pseudomonas aeruginosa* PGPR2 was purified by preparative high-performance liquid chromatography. It was found through antibacterial experiment that the purified compound showed antifungal activity against *Macrophomina phaseolina* and other phytopathogenic fungi. Subsequently, the spectral data suggests that the antifungal compound is 3,4-dihydroxy-*N*-methyl-4-(4-oxochroman-2-yl) butanamide, with the chemical formula C14H17NO5 and a molecular mass of 279. The compound has a 4-chromanone backbone, and chromanone and other related ring systems have several interesting biological activities, including antifungal effects. In addition, a series of sulfonamides have antibacterial and antifungal activities [128,129]. However, this article is the first to report the biological production of a chromanone derivative with antifungal activity. The discovery of this new compound can lay a foundation for further research on the biological control mechanism of the bean root-knot nematode. Comparative and pangenomic analysis of the *Pseudomonas aeruginosa* PGPR2 genome will open a new research area.

Kaliannan Durairaj et al. [65] isolated two strains from mine soil microorganisms with strong inhibitory activity against five plant pathogens, *Burkholderia glumae* (KACC 10138), *Xanthomonas oryzae pv. Oryzae* (KACC 10208), *Pseudomonas syringae* (KACC 15105), *Pectobacterium carotovorum* (KACC 17004), and *Ralstonia solanacearum* (KACC 10718)); these strains were identified as *Pseudomonas aeruginosa* D4 and *Bacillus stratosphericus* FW3. Both strains are highly effective at producing antimicrobial compounds. They also produce protease, amylase, and chitinase biocatalysts, along with increased siderophore production and phosphate solubilization, which promote plant growth. The results showed that the local application of *Pseudomonas aeruginosa* and *Bacillus stratosphericus* to tomato plants significantly induced the expression of the specific defence gene PR-1a. The authors subsequently reported that the expression of PAL in tomato leaves treated with *Pseudomonas aeruginosa* (OD6000.1) for 6 h was 32 times greater than that in untreated leaves. Preliminary studies have indicated that *Pseudomonas aeruginosa* and *Bacillus stratosphericus* stimulate systemic acquired resistance (SAR) through salicylic acid (SA)- and phenylalanine ammonia lyase (PAL)-dependent pathways.

Entomopathogenic fungi (EPF) are microorganisms that can infect insects to prevent and control insect infestation [130,131]. One of the more common EPF, *Beauveria bassiana*, can infect and kill a variety of insects, including pine caterpillars, corn borers, thrips, whiteflies, aphids, and planthoppers [132,133,134,135,136], and plays an important role in pest control [137]. It can be used in bait or spray form to control or kill housefly adults but has little effect on larvae (maggots) [134]. In this study, *Pseudomonas aeruginosa* was found to be the real cause of the resistance of housefly larvae to *Beauveria bassiana* infection. Shumin Wang et al. [66] isolated the strain *Pseudomonas aeruginosa* Y12, which has anti-*Beauveria bassiana* activity, from the gut of housefly larvae. The *Pseudomonas aeruginosa* Y12 strain was compared with *Pseudomonas aeruginosa* P18, which was isolated from wastewater but did not show anti-*Beauveria bassiana* activity. Through whole-genome sequencing and metabolomics analysis, we investigated the mechanism of the different antibacterial activities of two strains of *Pseudomonas aeruginosa*. After the study, it was found that the content of secondary metabolites was different between Y12 and P18, and six bioactive compounds that might be related to the antimicrobial activity of Y12 were identified. And the content of antibacterial substances of strain Y12 was higher than that of strain P18. In larval experiments, *Pseudomonas aeruginosa* colonized the larval breeding environment and released metabolic compounds that are toxic to *Bacillus bassiana*, thereby protecting housefly larvae from infection by entomopathogenic fungi. This study revealed that *Pseudomonas aeruginosa* Y12 has an important effect on housefly larvae and helps houseflies resist invasion by *Bacillus glossoides*. This study provides a research basis and broad application prospects for the possible role of *Pseudomonas aeruginosa* in the invasion of insect-borne fungi.

Francis Kwaku Dzideh Amankwah et al. [68] isolated *Pseudomonas aeruginosa* DO5 with broad-spectrum antibacterial activity from the Dompoase landfill site, and its ethyl acetate extract was found to have antibacterial activity against a variety of pathogens, including *Staphylococcus aureus* (ATCC25923), *Enterococcus faecalis* (ATCC 29212), *Escherichia coli* (ATCC 25922), and other pathogens. In agar diffusion experiments, the zones of inhibition of the extract of isolate DO5 ranged between 11.67 ± 0.23 and 21.50 ± 0.71 mm. The MIC and MBC recorded for the DO5 extract ranged from 3.13 mg/mL to 25.0 mg/mL and from 6.25 mg/mL to 50.0 mg/mL, respectively. The authors subsequently identified 9 compounds with reported antibacterial effects, including E-15-heptadecenal, 1-docosene, 3-eicosene, 1-eicosanol, 1-nonadecene, 1-hexadecanol, pyrrolizidine (pyrrolo) 1,2-apyrazine-1,4-dione, 1-hexadecene, and 1-heptacosanol, via GC–MS analysis. Thus, DO5 metabolites could be developed into potent antibiotics for infection treatment.

The strain PUPa3 was isolated from rice rhizosphere soil by R. Sunish Kumar et al. [70] and was identified as *Pseudomonas aeruginosa* by biochemical tests and 16S rDNA sequence comparison. The strain has a broad spectrum of antifungal activity against plant pathogenic fungi. The production of indole-3-acetic acid (IAA), siderophores, phosphatase, and protease in PUPa3 was determined. The antifungal metabolite produced by PUPa3 was identified as phenazine-1-carboxamide (PCN) based on NMR and MS data. The strain PUPa3 also exhibited several traits that promoted plant growth but did not exhibit harmful rhizobium traits. In summary, the new strain of *Pseudomonas aeruginosa* PUPa3 from the rhizosphere soil of rice was demonstrated to be an effective biocontrol agent.

*Pseudomonas aeruginosa* RM-3 was isolated from Minaxi and Jyoti Saxena [71]. In double-plate and liquid culture experiments, the strain showed good antibacterial activity against *Macrophomina phaseolina* and *Dreschlera graminae*. The authors subsequently observed through light and scanning electron microscopy the morphological changes in the mycelia, such as perforation, fragmentation, swelling, atrophy, and lysis, after bacterial treatment. The strain was also shown to produce siderophores and hydrogen cyanide (HCN). In addition, this strain also produces the extracellular chitinase enzyme and an important antibiotic, phenazine. In a pot experiment, it was observed that seed bacterization with *Pseudomonas aeruginosa* RM-3 significantly (*p* < 0.05) promoted the growth of mung bean seedlings. It was also able to colonize the rhizosphere of plants and reduce the percentage of disease incidence in *M. phaseolina*-infested soil by 82.8%, thereby increasing production. The secondary metabolites produced by *Pseudomonas aeruginosa* RM-3 have shown potential for promoting plant growth and significantly inhibiting soil-borne plant pathogenic fungi both in vitro and in vivo.

Nazneen Bano and Javed Musarrat [72] isolated *Pseudomonas aeruginosa* NJ-15 from soil; this strain produces large amounts of indole acetic acid (IAA) in a culture medium supplemented with tryptophan. In addition, this strain can also produce a large amount of siderophores and hydrogen cyanide (HCN). And with iron stimulation, the production of HCN also increases. The strain NJ-15 inhibits the growth of a wide range of phytopathogenic fungi, including *Fusarium oxysporum*, *Trichoderma herizum*, *Alternaria alternata*, and *Macrophomina phaseolina*.

Viviane F Cardozo et al. [74] used methicillin-resistant *Staphylococcus aureus* (MRSA) as an indicator bacterium to study the antibacterial activity of *Pseudomonas aeruginosa*-derived compounds against MRSA strains. The antibacterial substances were purified and identified. The authors subsequently isolated phenazine-1-carboxyamide from the purified component and reported that it had slight antibacterial activity against MRSA. To date, no studies have shown that phenazine-1- carboxamide has an antibacterial effect on MRSA. The studies have only reported that phenazine has antibacterial activity against major rice pathogens, such as *Rhizoctonia solani* and *Xanthomonas oryzae* pv. Oryzae. Moreover, the authors reported that other portions of this component have antibacterial effects or synergistic antibacterial effects. The authors conclude that the other components are organohalogen compounds, but specific structures have not been determined. By scanning electron microscopy, the authors reported that antimicrobial substances caused morphological changes in the cell wall of the MRSA strain. The authors also reported that the combination of phenazine-1-carboxamide and silver nanoparticles produced by *F. oxysporum* had synergistic effects, decreasing the MIC value of phenazine up to 32-fold. Studies involving synergistic effects have been very important for antibacterial therapy, mainly for multi-resistant bacteria. The results of this study suggest that the use of a secondary metabolite from bacteria such as *Pseudomonas aeruginosa* could be effective against MRSA strains that cause diseases in humans and other animals. This compound may be a good alternative for treating and controlling infections caused by MRSA.

*Pseudomonas aeruginosa* PF23 showed high salt tolerance and extracellular polysaccharide (EPS) production under 2000 mM NaCl. Sakshi Tewari and Naveen Kumar Arora reported that the yield of EPS increased with increasing salt concentration [76]. At low salt concentrations (up to 500 mM NaCl), PF23 contained 0.901 g/L EPS, which consisted mainly of glucose and galactose as its components. EPS has strong biological control potential against *Mycoplasma phaseolina*. Whereas a further increase in salinity (up to 2000 mM NaCl) resulted in glucose, rhamnose, mannose, and trehalose as major constituents of EPS, at high concentrations, EPS behaved as an osmoprotective or stress ameliorating metabolite, and when introduced in saline soil, it served as a plant growth promoter along with a seed biopriming agent. The authors conducted plant and in vivo studies with sunflower as the experimental crop and reported that PF23 can promote plant growth and yield and has significant biocontrol potential against the phytopathogen *Macrophomina phaseolina* (under saline conditions). This study revealed that EPS, a metabolite of *Pseudomonas aeruginosa* PF23, can increase the yield of sunflower crops in semiarid areas and minimize the incidence of sunflower carbon rot.

In the present study, Ankita Chopra et al. [77] isolated the strain *Pseudomonas aeruginosa* RTE4 from tea rhizosphere soil collected from the Rosekandy Tea Garden, Cachar, Assam, which was evaluated for various plant-growth-promoting attributes. The authors found that the strain RTE4 produces indole acetic acid, hydrolytic enzymes, and solubilized tri-calcium phosphate during the growth process, and evaluated the antibacterial activity and biocontrol ability of strain RTE4 against *Corticium invisium* and *Fusarium solani* and *Xanthomonas campestris*. During the initial screening, the strain RTE4 produced biosurfactants (BS) in a positive manner. Detailed analytical characterisation by the authors using TLC, FTIR, NMR, and LCMS techniques revealed that strain RTE4 grew in M9 medium containing glucose (2% *w*/*v*) produce dirhamnolipid BS. This BS reduced surface tension of phosphate buffer saline from 71 mN/m to 31 mN/m with a critical micelle concentration of 80 mg/L. The purified strain RTE4 showed minimum inhibitory concentration of 5, 10, and 20 mg/mL against *Xanthomonas campestris*, *Fusarium solani*, and *Corticium invisium*, respectively. In addition, the strain RTE4 produces di-rhamnolipid BS and displays several other characteristics that support plant growth. Consequently, the strain RTE4 possesses a wide range of plant-growth-promoting rhizobacterial (PGPR) characteristics, and the BS compounds it produces have a wide range of potential uses in agriculture as biocides.

## 3. Conclusions

With the continuous development of the discovery of natural bioactive metabolites, *Pseudomonas aeruginosa* is bound to play an important role. Although the research history of *Pseudomonas aeruginosa* biocontrol strains is relatively limited, the application of most *Pseudomonas aeruginosa* biocontrol strains in biological control is limited to live bacteria control. However, in recent years, the powerful antibacterial activity of *Pseudomonas aeruginosa* has been widely studied by researchers. A series of inhibitory secondary metabolites with novel structures and diverse functions were isolated and identified. The secondary metabolites of *Pseudomonas aeruginosa* are rich in diverse antibacterial substances and have good antibacterial activity, which provides great hope for the development of effective antibacterial drugs to prevent and control the microbial infections by drug-resistant pathogens. However, at the same time, many problems of development still have yet to be solved. One example is the problem of composition analysis; *Pseudomonas aeruginosa* secondary metabolites are diverse, but current technology is unable to analyze all the metabolites, and it is still relatively difficult to sufficiently separate and purify all substances. A second limitation relates to low yields and environmental problems; only relying on fermentation to improve the yields of secondary metabolites is a very limited approach, but to date, genetic improvements, condition optimization, and other technologies have been improved. Moreover, the loss of organic reagents during the extraction process has also had a small effect on the environment. These problems are also important directions for future research. The more important problem also includes the application of antibacterial substances, and the products developed with the secondary metabolites of *Pseudomonas aeruginosa* as the main compounds are still relatively few. The instability of the control effect and the rapid growth of drug-resistant bacteria make it difficult to progress in agricultural and clinical applications. At present, structural modification or chemical modification is still needed to overcome drawbacks and increase the stability of antibacterial substances.

This paper calls attention to the fact that different *Pseudomonas aeruginosa* strains have the potential to produce one or several antimicrobial metabolites at the same time. Moreover, they have good antibacterial activity against both pathogenic bacteria and pathogenic fungi. The metabolites of *Pseudomonas aeruginosa* have led to great achievements in the fields of public safety, medical protection, biological control, and drug development. The diversity of these compounds highlights their importance in future research, advancements and practical applications across various industries. In the field of biotechnology development, an increasing number of researchers are still exploring the vast array of potentially extremely valuable *Pseudomonas aeruginosa* metabolites.

## Figures and Tables

**Table 1:** Antimicrobial metabolites from *Pseudomonas aeruginosa*.

Source	Strain Name	Antibiotic Substance	Inhibition of Pathogenic Bacteria	References
Water samples	*Pseudomonas aeruginosa* HRW.1S3	Phenazine 1,6-dicarboxylic acid (PDC)	*Bacillus subtilis*	[48]
			*Bacillus thuringiensis* KPWP1	
			*Bacillus cereus* MSMS1	
			*E. coli*	
Rice inter-roots	*Pseudomonas aeruginosa* MML2212	Phenazine1-carboxamide (PCN)	*Rhizoctonia solani*	[49]
			*Xanthomonas oryzae pv. oryzae.*	
Laboratory separation	*Pseudomonas aeruginosa* UWI-1	Tris(1H-indol-3-yl) methylium, Bis(indol-3-yl) phenylmethane Indolo (2, 1b) quinazoline-6, 12 dione	*Bacillus Cereus* *Listeria Monocytogenes* *Corynebacterium Diphtheria* *MR-Staphylococcus Aureus* *Streptococcus Pyogenes* *Escherichia Coli* *Salmonella Enteritidis* *Neisseria Meningitide* *Klebsiella Oxytoca* *Haemophilus Influenza* (bis(indol-3-yl) phenylmethane inactive against Gram-negative bacteria)	[50]
Laboratory separation	*Pseudomonas aeruginosa* UICC B-40	(2E,5E)- phenyltetradeca-2,5-dienoate	*Staphylococcus aureus* ATCC 25923 *Bacillus cereus* ATCC 10876	[51]
Oil-contaminated soil sample	*Pseudomonas aeruginosa* 47T2	Mixture of rhamnolipids: Rha-Rha-C8-C10 RhaC10-C8/Rha-C8-C10 Rha-Rha-C8-C12:1 Rha-Rha-C10-C10 Rha-Rha-C10-C12:1 Rha-C10-C10 Rha-Rha-C10-C12/RhaRha-C12-C10 Rha-C10-C12:1/Rha-C12:1-C10 Rha-Rha-C12:1C12 Rha-Rha-C10-C14:1 Rha-C10-C12/Rha-C12-C10	*Serratia marcescens* *Enterobacter aerogenes* *Klebsiella pneumoniae* *Staphylococcus aureus* *Staphylococcus epidermidis* *Bacillus subtilis* *Chaetonium globosum* *Penicillium funiculosum* *Gliocadium virens* *Fusarium solani*	[52]
Rice inter-roots	*Pseudomonas aeruginosa* BRp3	Siderophores Rhamnolipids 4-hydroxy-2-alkylquinolines (HAQs) 2,3,4-trihydroxy-2-alkylquinolines 1,2,3,4-tetrahydroxy-2-alkylquinolines	*Xanthomonas oryzae pv. Oryzae* *Fusarium* spp.	[43]
Tropical estuarine habitats	*Pseudomonas aeruginosa* MBTDCMFRI Ps04	4-Hydroxy-11-methylpentacyclo [11.8.0.02,3.011,12.016,17] Henicosa-1,3,5,8(9),17-penten-14-one	*Aeromonas* *Vibrio*	[53]
Soil sample	*Pseudomonas aeruginosa* HBD-12	1-hydroxy-9,10-diazaphenanthrene 3-hydroxy-9,10-dihydrodiazaphenanthrene	*Escherichia coli* *Bacillus thuringiensis* *Penicillium italicum* *Penicillium fingerlingi*	[54]
Fruit sample	*Pseudomonas aeruginosa* LV	Phenazine-1-carboxylic acid (PCA) Phenazine-carboxamide (PCN) Indol-3-one (IND) Organocopper antimicrobial compound	*Phakopsora pachyrhizi*	[29]
Wastewater sample	*Pseudomonas aeruginosa* ST5	Mixture of rhamnolipid: Rha–C12–C10/Rha–C10–C12 Rha– C10–C10 Rha–C10–C8/Rha–C10–C8 Rha–Rha–C12–C10/RhaRha–C10–C12 Rha–Rha–C10–C10 Rha–Rha–C10–C8/Rha–Rha–C10–C8	*Escherichia coli* ATCC 417373 *Klebsiella pneumoniae* *Salmonella enterica* *Staphylococcus aureus* ATCC 25923 *Bacillus cereus* ATCC 10876 *Bacillus cereus* *Cryptococcus neo-formans* CAB1063 *Candida albicans* 8911	[55]
Plant-endophyte bacteria (lily bulb)	*Pseudomonas aeruginosa* Ld-08	3,9-dimethoxypterocarpan Cascaroside B Dehydroabietylamine Epiandrosterone Nocodazole Oxolinic acid Pyochelin Rhodotulic acid 9,12-octadecadienoic acid Dipeptides Tripeptides Ursodiol Venlafaxine	*Fusarium oxysporum,* *Botrytis cinerea* *Botryosphaeria dothidea* *Fusarium fujikuroi*	[56]
Laboratory separation	*Pseudomonas aeruginosa* PA06	Phenazine-1-carboxylic acid (PCA)	*Staphylococcus aureus* ATCC 25923	[57]
Oceanic sediment	*Pseudomonas aeruginosa* PA31x	Phenylamine-1-carboxylic acid (PCA)	*Vibrio anguillarum* C312 *Phytophthora nicotianae* JM1	[58]
Laboratory separation	*Phytophthora nicotianae* RLimb	Uncharted	*Bacillus cereus* *Streptococcus uberis* *Pseudomonas* sp. *Vibrio parahaemolyticus*	[59]
Rice inter-roots	*Pseudomonas aeruginosa* PA1201	Shenazinomycin phenazine-1-amide (PCN)	*Fusarium oxysporum*	[60]
Laboratory separation	*Pseudomonas aeruginosa* PM12	3-hydroxy-5-methoxy benzene methanol (HMB) Eugenol Tyrosine	*Fusarium oxysporum*	[61]
Patient sputum	*Pseudomonas aeruginosa*	Isocitrate lyase and malate synthetase Catalase and thioredoxin	*Aspergillus fumigatus*	[62]
The root of Millettia speciosa	*Pseudomonas aeruginosa* 2016NX1	1-hydroxyphenazine	*Salmonella* sp. *Klebsiella oxytoca* *Shigella castellani* *Salmonella typhi* *Bacillus anthracis* *Cochliobolus miyabeanus* *Diaporthe citri* *Rhizoctonia solani* *Phytophthora parasitica var. nicotianae* *Exserohilum turcicum*	[63]
The root surface of a mungbean plant	*Pseudomonas aeruginosa* PGPR2	3,4-dihydroxy-*N*-methyl-4-(4-oxochroman-2-yl)	*Macrophomina phaseolina*	[64]
Mine soil microorganisms	*Pseudomonas aeruginosa* D4	Protease, amylase Chitinase biocatalysts	*Burkholderia Glumae* KACC 10138 *Xanthomonas oxyzae pv.Oryzae* KACC 10208 *Pseudomonas syringae* KACC 15105 *Pectobacterium carotovorum* KACC 17004 *Ralstonia solanacearum* KACC 10718	[65]
The gut of housefly larvae	*Pseudomonas aeruginosa* Y12	Phenazine-1,6-dicarboxylic acid (PDC) Pyocyanin (PYO) Demeclocycline-HCl 2-hydroxy-4-methoxyacetophenone-5-sulfate Sulfamethoxypyridazine Orientin-7-O-sulfate Terconazole	*Beauveria bassiana*	[66]
Laboratory separation	*Pseudomonas aeruginosa* PA14	Four proteins (DVUA0018, DVUA0034, DVUA0066, and DVUA0084)	*Desulfovibrio vulgaris*	[67]
The landfill site	*Pseudomonas aeruginosa* DO5	E-15-heptadecenal 1-docosene 3-eicosene 1-eicosanol 1-nonadecene 1-hexadecanol Pyrrolizidine (pyr-rolo) 1,2-apyrazine-1,4-dione 1-hexadecene 1-heptaco-sanol	*Staphylococcus aureus* ATCC25923 *Enterococcus faecalis* ATCC 29212 *Escherichia coli* ATCC 25922 *Pseudomonas aeruginosa* ATCC 4853 *Streptococcus pyogenes* *Klebsiella pneumoniae* *Proteusmirabilis* *Vibrio cholera* *Salmonella typhi* *Candidaalbicans.*	[68]
Laboratory separation	*Pseudomonas aeruginosa* ID 4365	PyocyaninPhenazine-1-carboxylic	*Aspergillus niger* *Colletotrichum falcatum* *Colletotrichum capsicum* *Fusarium oxysporum* *Sclerotium rolfsii.*	[69]
Laboratory separation	*Pseudomonas aeruginosa* PUPa3	Phenazine-1-carboxamide (PCN) indole-3-acetic acid (IAA)	*Pestalotia theae* *Sarocladium oryzae* *Colletotrichum capsica* *Colletotrichum capsica* *Colletotrichum gleosporoides*	[70]
The root system of plants	*Pseudomonas aeruginosa* RM-3	Siderophore Hydrogen cyanide (HCN)	*Macrophomina phaseolina* *Dreschlera graminae*	[71]
Soil samples	*Pseudomonas aeruginosa* NJ-15	Siderophore Hydrogen cyanide (HCN)	*Fusarium oxysporum* *Trichoderma herizum* *Alternaria alternata* *Macrophomina phasiolina*	[72]
Soil samples	*Pseudomonas aeruginosa* SD12	1-hydroxyphenazine	*Alternaria alternata* *Bipolaris australiensis* *Colletotrichum acutatum* *Fusarium oxysporum* *Alternaria solani* *Colletotrichum acutatum*	[73]
Laboratory separation	*Pseudomonas aeruginosa*	Phenazine-1-carboxamide (PCN) Organohalogen compounds	*Methicillin-resistant Staphylococcus aureus*	[74]
Soil samples	*Pseudomonas aeruginosa* GC-B26	Phenazine-1-carboxylic	*Colletotrichum orbiculare* *Phytophthora capsica* *Pythium ultimum*	[75]
The root system of plants	*Pseudomonas aeruginosa* PF23	Extracellular polysaccharide (EPS)	*Macrophomina phaseolina*	[76]
The root system of plants	*Pseudomonas aeruginosa* RTE4	Indole acetic acid Hydrolytic enzymes Solubilize tri-calcium phosphate Biosurfactant	*Corticium invisium* *Fusarium solani* *Xanthomonas campestris* *Staphylococcus aureus*	[77]

## Data Availability

This article is a review and does not report original data. The data supporting this review are from previously reported studies and datasets, which have been cited.
